# Genomic Prediction of Single Crosses in the Early Stages of a Maize Hybrid Breeding Pipeline

**DOI:** 10.1534/g3.116.031286

**Published:** 2016-09-19

**Authors:** Dnyaneshwar C. Kadam, Sarah M. Potts, Martin O. Bohn, Alexander E. Lipka, Aaron J. Lorenz

**Affiliations:** * Department of Agronomy and Horticulture, University of Nebraska, Lincoln, Nebraska 68583; † Department of Crop Sciences, University of Illinois at Urbana-Champaign, Urbana, Illinois 61801

**Keywords:** hybrid breeding, genomic prediction, genotyping by sequencing, general combining ability, specific combining ability, GenPred, Shared Data Resources, Genomic Selection

## Abstract

Prediction of single-cross performance has been a major goal of plant breeders since the beginning of hybrid breeding. Recently, genomic prediction has shown to be a promising approach, but only limited studies have examined the accuracy of predicting single-cross performance. Moreover, no studies have examined the potential of predicting single crosses among random inbreds derived from a series of biparental families, which resembles the structure of germplasm comprising the initial stages of a hybrid maize breeding pipeline. The main objectives of this study were to evaluate the potential of genomic prediction for identifying superior single crosses early in the hybrid breeding pipeline and optimize its application. To accomplish these objectives, we designed and analyzed a novel population of single crosses representing the Iowa Stiff Stalk synthetic/non-Stiff Stalk heterotic pattern commonly used in the development of North American commercial maize hybrids. The performance of single crosses was predicted using parental combining ability and covariance among single crosses. Prediction accuracies were estimated using cross-validation and ranged from 0.28 to 0.77 for grain yield, 0.53 to 0.91 for plant height, and 0.49 to 0.94 for staygreen, depending on the number of tested parents of the single cross and genomic prediction method used. The genomic estimated general and specific combining abilities showed an advantage over genomic covariances among single crosses when one or both parents of the single cross were untested. Overall, our results suggest that genomic prediction of single crosses in the early stages of a hybrid breeding pipeline holds great potential to redesign hybrid breeding and increase its efficiency.

Contemporary hybrid breeding programs are based on the pure-line method of corn breeding proposed by Shull (1909). This method includes the development of inbreds by self-pollination, followed by evaluation of selected inbreds for single-cross performance when crossed to other inbreds. A major challenge with this method is achieving adequate testing of the inbreds to evaluate performance in single-cross combinations (Hallauer *et al.* 1988). In maize, heterotic groups are well defined, and single crosses are almost exclusively made between heterotic groups. The fullest assessment of single-cross performance in maize, therefore, would be a complete factorial mating design achieved by making all between-heterotic groups single crosses. This would provide complete information on both general combining ability (GCA) and specific combining ability (SCA) (Comstock and Robinson 1948). However, a full factorial design among inbreds can be cost prohibitive, as advanced hybrid breeding programs typically have many inbreds to evaluate, making the number of all possible single crosses extremely large. For this reason, predicting single-cross performance has always been a major issue for hybrid breeding programs (Schrag *et al.* 2009).

Several approaches have been used to evaluate the genetic merit of inbreds for single-cross performance with variable success. These approaches include inbred *per se* performance, performance when crossed to testers (topcross test), best linear unbiased prediction (BLUP) using pedigrees, and molecular marker-assisted prediction. Many of these approaches have been reviewed in detail elsewhere (Schrag *et al.* 2009; Smith and Betrán 2004). *Per se* performance of inbreds is typically found to be a very poor predictor of single-cross performance, especially for traits such as grain yield (GY), where strong dominance effects underlie the genetic variance (Hallauer 1977; Love and Wentz 1914; Smith 1986). A topcross test is an established and simple approach to assess the genetic worth of inbreds in single-cross combinations (Jenkins and Brunson 1932). However, topcross evaluation of a large number of inbreds is difficult (Albrecht *et al.* 2011), and selections based on single-cross performances are carried out in later stages, increasing the time required for commercial hybrid development. Bernardo (1996a) showed that pedigree-based BLUP is useful for prediction of untested single crosses. He used pedigree-based covariance matrices among tested and untested single crosses to obtain BLUPs for untested single crosses. The correlations between observed and predicted performance were moderate (0.43–0.76) for single crosses where both parents were tested in single-cross combinations. However, correlations were severely decreased when one or both of parents were untested (Bernardo 1996b).

The relationship between genetic distance (GD) of parental inbreds, measured by molecular markers, and heterosis has been extensively studied in maize. While it is possible to predict single-cross performance using marker-based GD for hybrid sets composed of both intra- and inter-heterotic group single crosses, correlations for predicting interheterotic group single crosses only were reported to be very low (Lee *et al.* 2007; Melchinger 1999). Two possible causes of these low prediction accuracies include (1) loose association between heterotic quantitative trait loci (QTL) and the molecular markers used to estimate GD, and (2) opposite linkage phases between the QTL and marker alleles across heterotic groups (Bernardo 1992; Charcosset *et al.* 1991). Commercial hybrids consist of only interheterotic group single crosses, making them the only type relevant for prediction in breeding programs. In a modified approach, prediction of single-cross performance and SCA based on only significant markers was suggested (Vuylsteke *et al.* 2000), but this approach was found to be inferior to an established GCA method. Also, extending the GCA predictions with SCA estimates from associated markers did not improve the prediction accuracy (Schrag *et al.* 2006, 2007).

Genomic prediction is an approach that uses markers to predict the genetic value of complex traits in progeny for selection and breeding (Meuwissen *et al.* 2001). When genomic predictions are used to make selections, it is referred to as genomic selection (GS). The primary difference between GS and traditional forms of marker-assisted selection (MAS) is the simultaneous use of a large number of markers distributed genome-wide, as opposed to a small set of markers linked to QTL (Heffner *et al.* 2009). Implementation of genomic prediction and selection requires the development of training (calibration) sets consisting of individuals that have been both phenotyped and genotyped, followed by model calibration. A whole suite of genomic prediction models have been developed, each deploying different strategies to estimate genome-wide marker effects or model genomic relationships between individuals (de los Campos *et al.* 2013).

Recent results from simulation and experimental studies have indicated the usefulness of genomic prediction models to predict hybrid performance in maize (Albrecht *et al.* 2011, 2014; Jacobson *et al.* 2014; Massman *et al.* 2013; Riedelsheimer *et al.* 2012; Technow *et al.* 2012, 2014; Windhausen *et al.* 2012). However, most of the experimental studies were focused on prediction of topcross performance under a single tester scenario (Albrecht *et al.* 2011, 2014; Jacobson *et al.* 2014; Riedelsheimer *et al.* 2012; Windhausen *et al.* 2012). Experimental studies on genomic prediction of single-cross performance have been based on historical data consisting of established inbred parents with mixed and complex ancestry (Massman *et al.* 2013; Technow *et al.* 2014). These studies used covariances among tested and untested single crosses estimated from realized genomic relationship matrices to predict the performance of untested single crosses. The prediction accuracies were high, often exceeding 0.75, even when both parents of the single cross were untested.

Identification of superior single crosses early in the hybrid breeding pipeline would be beneficial to developing commercial hybrids more quickly. The current practice of initial selection among available inbreds based on their topcross performance, followed by evaluation of single crosses made among selected inbreds, increases time required for commercial hybrid development. Moreover, this approach does not allow evaluation of all possible single-cross combinations among available inbreds. It is important, therefore, to study the potential of genomic prediction of early-stage single crosses (*i.e.*, single crosses between sets of random inbreds derived from each heterotic group).

With this in mind, the main objective of this study was to evaluate the potential of genomic prediction for identifying superior single crosses early in the breeding pipeline. Also, the effect of model and training set composition on single-cross prediction accuracy was evaluated. To accomplish these objectives, we designed and analyzed a novel population of single crosses. The parental recombinant inbred lines (RILs) and doubled haploid lines (DHLs) were randomly selected from three Iowa Stiff Stalk synthetic (SSS) and three non-Stiff Stalk synthetic (NSS) biparental populations. All single crosses, therefore, represented the SSS/NSS heterotic pattern commonly used in the development of North American commercial maize hybrids. All RILs and DHLs were genotyped using genotyping by sequencing (GBS) (Elshire *et al.* 2011); an affordable genotyping option which is critical to the routine use of these methods in a breeding program.

## Materials and Methods

### Germplasm

**Figure 1:**
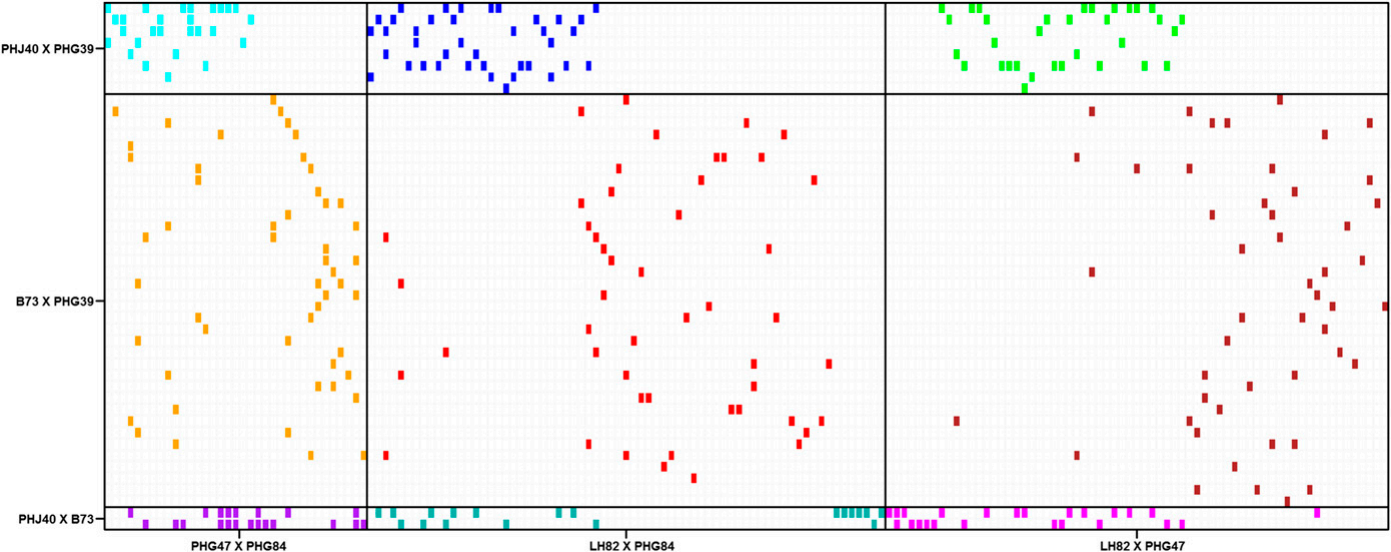
Crossing scheme between RILs or DHLs derived from three biparental families representing the SSS (*y*-axis) and NSS (*x*-axis) heterotic groups. Colored boxes indicate the presence while unfilled boxes indicate absence of a particular single cross. Bold lines delineate single-cross families.

Three SSS inbred parents (PHG39, PHJ40, and B73) and three NSS inbred parents (LH82, PHG47, and PHG84) were used to create six biparental families by making each of the three possible crosses between the three SSS inbreds, and also between the three NSS inbreds (Supplemental Material, Figure S1). The parents were selected to be both genetically diverse and superior in GCA for GY under high planting density (Mansfield and Mumm 2014). A total of 217 lines were developed from crosses between these parents. Approximately 10% of these lines were RILs and 90% were DHLs. RILs and DHLs will hereafter be referred to collectively as “inbred progenies.” The number of inbred progenies in each of the six biparental families ranged from two to 69 (Table 1). Random crosses among the inbred progenies were made between heterotic groups to produce 312 single crosses (Figure 1). Single crosses representing each biparental family were balanced to the extent possible while maximizing the number of inbred progenies used in the single crosses. Completely balanced representation was not achieved due to seed limitations and comparatively fewer inbred progenies available for certain biparental families. Single crosses were grouped into nine single-cross families, which we define as a group of single crosses created using inbred progenies from the same biparental family on each side of the heterotic pattern (Table 1). For example, a single cross with pedigree (PHJ40 × PHG39)DH-1/(PHG47 × PHG84)DH-1 belongs to the same single-cross family as a single cross with pedigree (PHJ40 × PHG39)DH-2/(PHG47 × PHG84)DH-2. The mean number of times an individual SSS inbred progeny was used in a cross was 6.9. The mean number of times an individual NSS inbred progeny was used in a cross was 1.8. Number of single crosses per single-cross family ranged from 19 to 51 (Table 1).

**Table 1:** Family designations of nine single-cross families and number of single crosses belonging to each of the nine families

	PHG47 × PHG84 (35)	LH82 × PHG47 (69)	LH82 × PHG84 (67)	Total
PHJ40 × PHG39 (8)	f1 (27)	f2 (39)	f3 (33)	99
B73 × PHG39 (36)	f4 (51)	f5 (49)	f6 (49)	149
PHJ40 × B73 (2)	f7 (21)	f8 (19)	f9 (24)	64
Total	99	107	106	312

Biparental families are listed in the row and column headings. The numbers in the parentheses indicate numbers of recombinant inbred lines (RILs) or doubled haploid lines (DHLs) in the biparental family or number of single crosses in each single-cross family. The total number of single crosses are displayed in 4th column (for SSS biparental families) and 4th row (for NSS biparental families).

### Field experiments

The 312 single crosses were evaluated at two locations in 2012 and three locations in 2013. Two locations were common between years. The locations were as follows: South Farms (Urbana, IL; 2012 and 2013), Maxwell Farms (Urbana, IL; 2012 and 2013), and Monmouth (IL; 2013 only). The five location–year combinations were defined as separate environments. The experimental design was an α(0, 1)-incomplete block design (Patterson and Williams 1976) with three replications at each environment. All trials were planted with an Almaco Seed Pro 360 planter set at 0.64 m row spacing and 4.46 m long row. Entries were grown in small plots consisting of two rows. Plots were overplanted by 15% to compensate for germination failure and later thinned to the target plant density of 116,000 plants/ha^–1^. All fields were controlled for weeds. Nitrogen (N) was applied before planting as 28% urea-ammonium nitrate at a rate of 336.4 kg/ha to all fields. Phosphorous and potassium were each applied at 112 kg/ha according to recommended levels determined by soil tests performed by the University of Illinois Crop Science Research and Education Center. Stand counts were recorded and plots with planting densities lower than 106,000 plants/ha^–1^ discarded. Additionally, issues with seed production resulted in fewer single crosses being planted at all locations in 2013 (South Farms, 260; Maxwell Farms, 259; and Monmouth, 258). Plots were machine harvested and data were recorded for several agronomic traits. For this study, data on GY, plant height (PH), and staygreen (SG) were used for downstream analyses. GY was converted to Mt/ha on a 155 g/kg moisture basis. PH was measured postanthesis on a single representative plant determined by visually surveying the entire plot before measurement. SG was evaluated visually as a percentage of total dry down, where a rating of 1 represented complete senescence and a rating of 10 represented fully green leaves.

### GBS

Five plants of each inbred progeny were germinated. A total of 0.1 g of tissue was sampled from leaf tips and pooled across the five plants. DNA was extracted using the Qiagen DNeasy Plant 96 kit following the DNeasy Plant Handbook. DNA samples were sent to the Institute for Genomic Diversity at Cornell University for GBS, where library construction and sequencing was performed as described by Elshire *et al.* (2011). Single nucleotide polymorphisms (SNPs) were scored from the raw sequence data using the TASSEL GBS Pipeline version 3.0 (Glaubitz *et al.* 2014). SNPs with >20% missing values and <5% minor allele frequency were removed from the dataset. Heterozygotes were treated as missing data. Missing data were replaced by the mean coded value for the marker (*i.e.*, naïve imputation). Of the markers remaining after filtration, markers that were polymorphic among both SSS and NSS inbred progenies were retained for analysis. The final marker data set consisted of 2296 high-quality SNPs. The distribution of SNPs among the 10 chromosomes of the B73 reference genome is displayed in Figure S2.

### Phenotypic data analysis

The phenotypic data were unbalanced due to missing observations. We used the following statistical model for the analysis of the data across the five environments:

$$y_{iklq}=\mu +g_{i}+e_{k}+{\left(ge\right)}_{ik}+r_{l\left(k\right)}+b_{q\left(kl\right)}+\varepsilon_{iklq},$$ (1)

where $y_{iklq}$ is the phenotypic observation for *i*^th^ single cross evaluated in the *k*^th^ environment in the *l*^th^ complete block (*i.e.*, replicate) and *q*^th^ incomplete block. The effects in the model are as follows: *μ* is the grand mean; $g_{i}$ represents effect of the *i*^th^ single cross; $e_{k}$ represents the effect of the *k*^th^ environment; ${\left(ge\right)}_{ik}$ represents the interaction effect between single cross and environment; $r_{l\left(k\right)}$ represents the effect of the *l*^th^ complete block nested within the *k*^th^ environment; $b_{q\left(kl\right)}$ represents the effect of the *q*^th^ incomplete block nested within the *l*^th^ complete block in the *k*^th^ environment; and $\varepsilon_{iklq}$ represents the residual. Environment and replication nested within environment effects were modeled as fixed effects while all other effects were treated as random. In model (1), $g_{i}\;∼\;N\left(0,\;I\sigma^{2}\right).$ Error and block variances were allowed to be heterogeneous among environments.

The above model was implemented using ASReml-R software (Butler *et al.* 2009) to obtain restricted maximum likelihood estimates of all variance components and solve the mixed linear model equations. Significance of the variance components was determined using likelihood ratio tests at 0.001 level of significance. The entry-mean heritability of each trait was computed according to Holland *et al.* (2003) as: $H^{2}=\sigma_{g}^{2}/\left(\sigma_{g}^{2}+\left(\sigma_{g\times e}^{2}/h_{k}\right)+\left(\sigma_{e}^{2}/h_{t}\right)\right),$ where, $\sigma_{g}^{2}$ represents the variance among single crosses, $\sigma_{g\times e}^{2}$ represents the variance of interaction effects of single crosses with environments, $\sigma_{e}^{2}$ is the residual variance, $h_{k}$ is the harmonic mean of number of observations per single cross within an environment, and $h_{t}$ is the harmonic mean of total number of observations per single cross. Similarly, model (1) was used to estimate the genetic variance and broad-sense heritability for an individual single-cross family. Finally, we calculated BLUPs of single crosses using model (1), which were used as validation to estimate genomic prediction accuracy in downstream analyses.

### Single-cross prediction methods

The linear model used for single-cross performance was as follows:

$$y_{ijklq}=\mu +f_{i}+m_{j}+s_{ij}+e_{k}+r_{l\left(k\right)}+b_{q\left(kl\right)}+{\left(fe\right)}_{ik}+{\left(me\right)}_{jk}+{\left(se\right)}_{ijk}+\varepsilon_{ijklq},$$ (2)

where $y_{ijklq}$ is the phenotypic observation on a single cross between the *i*^th^ and *j*^th^ inbred progeny, evaluated in the *k*^th^ environment in the *l*^th^ complete block and *q*^th^ incomplete block. The effects in the model are as follows: *μ* is the grand mean; $f_{i}$ and $m_{j}$ represents the GCA effects of the females (SSS inbred progenies) and males (NSS inbred progenies), respectively; $s_{ij}$ represents the SCA effect of the single cross; and ${\left(fe\right)}_{ik},{\left(me\right)}_{jk},\;\mathrm{and}\;{\left(se\right)}_{ijk}$ represent the interaction effects of respective terms with the *k*^th^ environment. The remaining terms were as described in model (1).

The random effect vectors f, m, and s were assumed to have the following multivariate normal (MVN) distributions: $f∼MVN\left(0,G_{f}\sigma_{GCA\_F}^{2}\right),$ $m∼MVN\left(0,G_{m}\sigma_{GCA\_M}^{2}\right),$ and $s∼MVN\left(0,\;S\sigma_{SCA}^{2}\right),$ where $G_{f}$ and $G_{m}$ were additive genomic relationship matrices of females and males, calculated as simple marker similarity coefficients. The dominance relationship matrix, S, was computed according to Bernardo (2002), using the corresponding elements from matrices $G_{f}$ and $G_{m}.$ Model (2) was implemented using ASReml-R (Butler *et al.* 2009).

We evaluated four methods to predict single-cross performance using model (2). Broadly, these methods can be grouped into two categories: (1) parent GCA and SCA effects, and (2) additive and dominance covariances among single crosses.

#### Method 1a: Parent GCA:

Performance of untested single crosses $\left({\hat{y}}_{u}\right)$ was predicted from the GCA of the corresponding parents, *i* and *j*, estimated from model (2) as:

$${\hat{y}}_{u}=\hat{\mu }+{\hat{f}}_{i}+{\hat{m}}_{j}.$$ (3)

GCA of females and/or males with no performance data were estimated from related inbred progenies using information from relatives through *G_m_* and/or *G_f_* in model (2).

#### Method 1b: Parent GCA and single-cross SCA:

Performance of untested single crosses $\left({\hat{y}}_{u}\right)$ was predicted using the sum of the parent GCA and SCA of the single crosses as:

$${\hat{y}}_{u}=\hat{\mu }+{\hat{f}}_{i}+{\hat{m}}_{j}+{\hat{s}}_{ij}.$$ (4)

As for the GCA effects, the SCA effects for untested single crosses were estimated using the dominance genomic relationship matrix in model (2).

#### Method 2a: Additive covariance among single crosses:

The performance of untested single crosses $\left({\hat{y}}_{u}\right)\;$ was predicted based on the covariance among tested and untested single crosses as:

$${\hat{y}}_{u}=C_{ut}C_{tt}^{-1}y_{t},$$ (5)

where $C_{ut}$ is the genetic covariance matrix of untested and tested single crosses, $C_{tt}$ is the phenotypic covariance matrix of the tested single crosses, and $y_{t}$ is a vector of tested single-cross BLUPs obtained from model (1). The elements of $C_{ut}$ and $C_{tt}$ were computed according to Bernardo (2002), using the genomic relationship matrices $G_{f}$ and $G_{m}.$ Briefly, let i and $i^{\prime }$ denote any two female (F) inbred progenies and j and $j^{\prime }$ any two male (M) inbred progenies. For a given pair of single crosses, (i × j) and ($i^{\prime }$ × $j^{\prime }$), the elements of $C_{ut}$ and the off-diagonal elements of $C_{tt}$ were calculated as ${\left(G_{f}\right)}_{ii^{\prime }}$ $\sigma_{GCA\_F}^{2}$ + ${\left(G_{m}\right)}_{jj^{\prime }}\sigma_{GCA\_M}^{2}.$ The diagonal elements of $C_{tt}$ were estimated as ${\left(G_{f}\right)}_{ii}$ $\sigma_{GCA\_F}^{2}$ + ${\left(G_{m}\right)}_{jj}\sigma_{GCA\_M}^{2}+\sigma_{\bar{X}}^{2},$ where $\sigma_{\bar{X}}^{2}$ was equal to $\sigma_{e}^{2}$ divided by the total number of observations for single cross (i×j). The estimates of $\sigma_{GCA\_F}^{2}$ and $\sigma_{GCA\_M}^{2}$ were obtained from model (2).

#### Method 2b: Additive and dominance covariance among single crosses:

The method described in 2a was extended by including dominance covariance among the tested and untested single crosses. Specifically, the elements of $C_{ut}$ and off diagonal elements of $C_{tt}$ were computed as ${\left(G_{f}\right)}_{ii^{\prime }}\sigma_{GCA\_F}^{2}$ + ${\left(G_{m}\right)}_{jj^{\prime }}\sigma_{GCA\_M}^{2}+{\left(G_{f}\right)}_{ii^{\prime }}{\left(G_{m}\right)}_{jj^{\prime }}\sigma_{SCA}^{2}.$ The diagonal elements of $C_{tt}$ were estimated as ${\left(G_{f}\right)}_{ii}$ $\sigma_{GCA\_F}^{2}$ + ${\left(G_{m}\right)}_{jj}\sigma_{GCA\_M}^{2}+{\left(G_{f}\right)}_{ii}{\left(G_{m}\right)}_{jj}\sigma_{SCA}^{2}+\sigma_{\bar{X}}^{2}.$ The estimates of $\sigma_{SCA}^{2}$ was obtained from model (2).

### Cross-validation and prediction accuracy estimation

**Figure 2:**
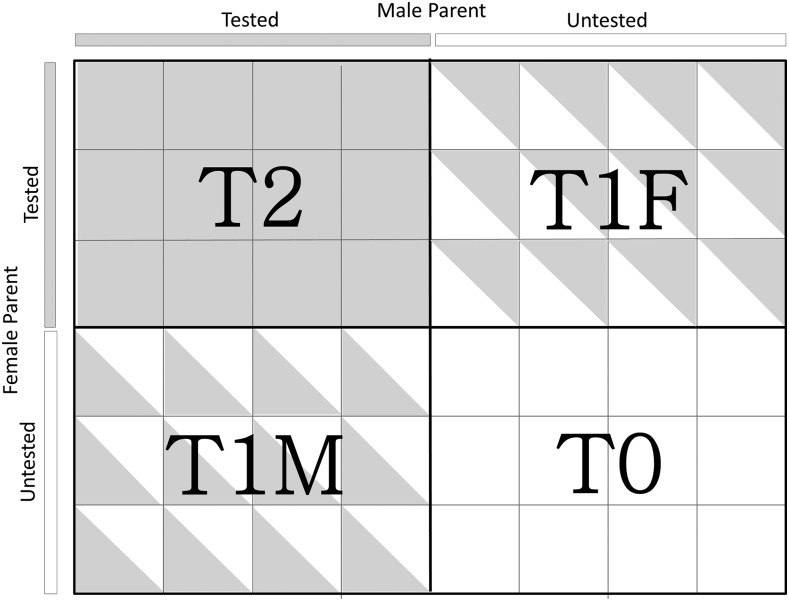
Schematic visualization of T2, T1F, T1M, and T0 cross-validation scenarios. Each small square represents one single cross. Completely filled squares (T2) indicate that both male and female parents of a single cross contained in the validation set were tested, half-filled squares indicate either the female (T1F) or male parent (T1M) of single cross contained in the validation set was tested, and unfilled squares (T0) indicate that neither parent of a single cross contained in the validation set was tested.

Accuracy of single-cross prediction was evaluated using leave-one-out cross-validation (LOOCV). LOOCV is a particular case of *k*-fold cross-validation with *k* = *n*. We chose LOOCV because the greater number of folds minimizes bias in the estimator (Kohavi 1995). Five different LOOCV scenarios involving varying degrees of relationship between training and validation set single crosses were considered (Figure 2). The cross-validation scenarios were as follows: (1) T2, both parents of a single cross contained in the validation set were tested; (2) T1F, only the female parent of a single cross contained in the validation set was tested; (3) T1M, only the male parent of a single cross contained in the validation set was tested; (4) T0, neither of the parents of a single cross contained in the validation set was tested; and (5) novel single-cross family, all single crosses belonging to one single-cross family were removed from the training set and thus formed the validation set. The conventional LOOCV was slightly modified to maintain constant training set size for each of the five cross-validation scenarios considered. The common maximum possible training set size across the five scenarios was 261. With this in mind, we decided to set the training set size to 250 for all five cross-validation scenarios in order to remove the confounding effect of population size. For the first four scenarios, the cross-validation is repeated such that each of the 312 single crosses was placed into the validation set exactly one time (*i.e.*, leave-one-individual-out cross-validation). For each of the 312 rounds, a random sample of 250 single crosses from the remaining single crosses was drawn without replacement and formed the training set. This was repeated 30 times to allow for sufficient resampling of the training set for a total of 9360 (30 × 312) resampled training sets. For each of the 30 repetitions, the predictions were integrated into a single vector and correlated with the phenotypic observations, as described below. For scenario 5, the cross-validation was repeated so that each of the nine single-cross families was entered into the validation set one time (*i.e.*, leave-one-family-out cross-validation). This was repeated 30 times by resampling 250 single crosses without replacement from the training set. The prediction accuracy, however, was evaluated only for the six largest families because size of the three families (f7, f8, and f9) was too small to accurately estimate correlation coefficients (Table 1).

The single-cross BLUPs returned from model (1) were treated as the observed single-cross performance and used for validation. Prediction accuracy was expressed as the Pearson’s correlation coefficient between the observed and predicted single-cross performance divided by the square root of the broad-sense heritability on an entry-mean basis (Dekkers 2007). The mean prediction accuracy across the 30 repetitions was reported. SEs of the prediction accuracy were calculated using the bootstrap method implemented in the *R* package, *boot* (Canty 2016). For each of the 30 repetitions, the predicted and observed values were resampled with replacement 200 times and the resulting distribution of 200 correlation coefficient estimates was used to estimate the bootstrap SE. The mean SE across the 30 repetitions was reported.

### Data availability

File S1 contains phenotypic data. File S2 contains BLUPs of single crosses. File S3 contains data for 2296 SNPs scored on SSS inbred progenies. File S4 contains data for 2296 SNPs scored on NSS inbred progenies. File S5 contains an additive genomic relationship matrix for SSS inbred progenies. File S6 contains an additive genomic relationship matrix for NSS inbred progenies. File S7 contains a dominance genomic relationship matrix for the single crosses.

## Results

### Variance components and broad-sense heritability

Variance among single crosses $\left(\sigma_{g}^{2}\right)$ was significantly different from zero (α=0.001) in the whole population as well as within individual single-cross families for all three traits (Table 2). For GY, the entry-mean heritability was 0.58 across the whole population of single crosses, and it ranged from 0.53 to 0.83 within the individual single-cross families. Similarly, for PH and SG, the entry-mean heritability was 0.89 and 0.81 in the whole population, respectively, and ranged from 0.88 to 0.91 and 0.67 to 0.80 within individual single-cross families, respectively. The sum of parent $\sigma_{GCA}^{2}$ was greater than $\sigma_{SCA}^{2}$ for all traits. The proportion of $\sigma_{SCA}^{2}$ was highest for GY, followed by PH and SG (Table 3).

**Table 2:** Mean, range, genetic variance, and broad-sense heritability estimates in whole population as well as individual single-cross families for grain yield (GY; Mt/ha), plant height (PH; cm), and staygreen (SG; 1–10 rating)

Trait	Statistic	Single-Cross Populations						
		Whole	f1	f2	f3	f4	f5	f6
GY	Mean	8.67	8.6	8.85	8.87	8.88	9.03	9.13
	Range	7.14–10.2	7.13–9.91	6.94–9.94	7.79–9.99	6.74–10.7	7.52–10.4	7.46–10.5
	$\sigma_{g}^{2}\pm \mathrm{SE}$	0.50 ± 0.07	0.9 ± 0.31	0.48 ± 0.18	0.25 ± 0.12	0.55 ± 0.19	0.51 ± 0.15	0.51 ± 0.16
	$H^{2}\pm \mathrm{SE}$	0.58 ± 0.04	0.80 ± 0.07	0.66 ± 0.09	0.53 ± 0.13	0.57 ± 0.10	0.71 ± 0.07	0.71 ± 0.07
PH	Mean	210.1	213.4	206.6	205.7	221.2	208.9	216.1
	Range	191–231	197–227	187–222	187–222	202–243	182–230	191–241
	$\sigma_{g}^{2}\pm \mathrm{SE}$	1.18 ± 0.1	0.71 ± 0.23	0.8 ± 0.22	0.95 ± 0.27	0.87 ± 0.21	0.9 ± 0.20	1.07 ± 0.24
	$H^{2}\pm \mathrm{SE}$	0.89 ± 0.01	0.88 ± 0.04	0.86 ± 0.04	0.90 ± 0.03	0.83 ± 0.04	0.90 ± 0.02	0.91 ± 0.02
SG	Mean	6.79	6.96	7.05	6.68	6.35	6.75	6.22
	Range	5.48–8.31	5.57–7.96	5.82–8.5	5.57–7.96	4.61–7.88	5.67–7.92	5.07–7.39
	$\sigma_{g}^{2}\pm \mathrm{SE}$	0.69 ± 0.07	0.36 ± 0.15	0.52 ± 0.16	0.26 ± 0.1	0.58 ± 0.14	0.38 ± 0.1	0.26 ± 0.07
	$H^{2}\pm \mathrm{SE}$	0.81 ± 0.02	0.67 ± 0.10	0.74 ± 0.07	0.68 ± 0.09	0.80 ± 0.04	0.78 ± 0.05	0.78 ± 0.05

**Table 3:** General combining ability variance of stiff stalk synthetic ($\sigma_{\mathrm{GCA\_F}}^{2}$) and non-stiff stalk ($\sigma_{\mathrm{GCA\_M}}^{2}$) inbred progenies and specific combining ability variance ($\sigma_{SCA}^{2}$) of single crosses between them.

Variance components	Grain yield	Plant height	Staygreen
$\sigma_{\mathrm{GCA\_F}}^{2}$	0.22**	28.66**	0.12**
$\sigma_{\mathrm{GCA\_M}}^{2}$	0.20**	34.48**	0.23**
$\sigma_{SCA}^{2}$	0.05**	2.6**	0.01**
$\frac{\sigma_{SCA}^{2}}{\left(\sigma_{\mathrm{GCA\_F}}^{2}+\sigma_{\mathrm{GCA\_M}}^{2}\right)}$	0.12	0.04	0.03

** Significant at α = 0.001

### Prediction accuracy for T2, T1F, T1M, and T0 scenarios

**Figure 3:**
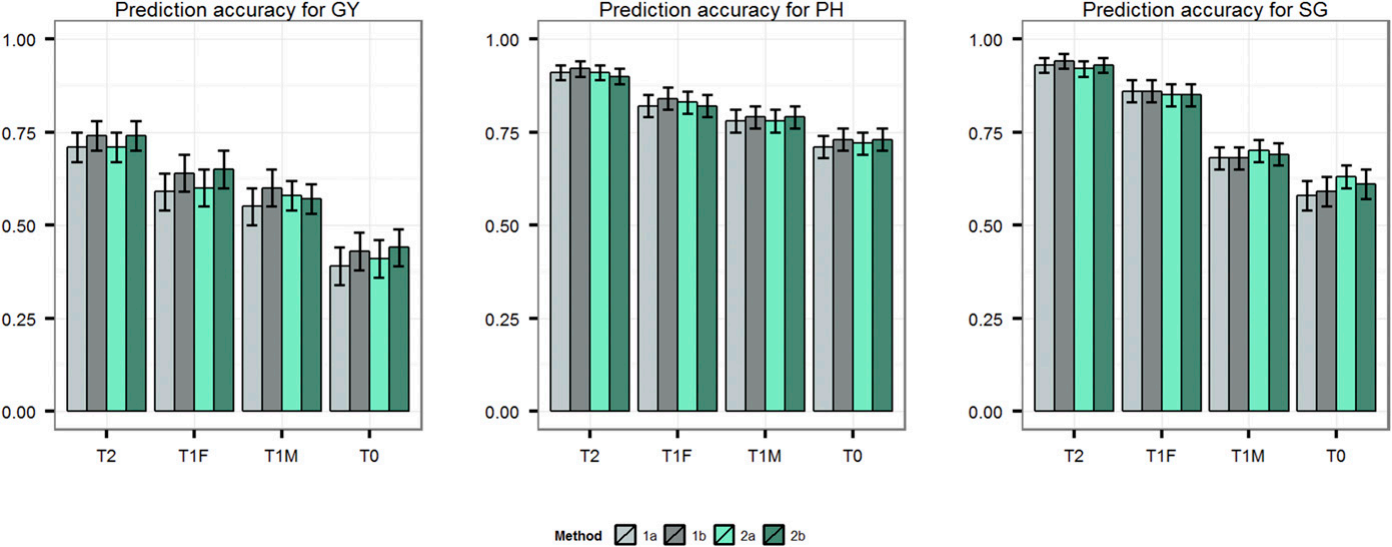
Prediction accuracy for T2, T1F, T1M and T0 cross-validation scenarios for traits grain yield (GY), plant height (PH) and staygreen (SG) obtained using the four methods 1a (Parent GCA), 1b (Parent GCA plus single-cross SCA), 2a (Additive genetic covariance among single crosses) and 2b (Additive plus dominance covariance among single crosses) as evaluated with training set of 250 and leave-one-individual-out cross-validation.

We first evaluated the prediction accuracy for T2, T1F, T1M, and T0 scenarios in the whole population using leave-one-individual-out cross-validation. Higher prediction accuracies were observed for SG and PH compared with GY for all scenarios (Figure 3). Prediction accuracies were highest for T2, followed by T1F, T1M, and T0. The four methods were similar in accuracy when applied to the T2 and T1F scenarios. However, methods 1a and 1b were mostly better than methods 2a and 2b for predicting single-cross performance in the T1M and T0 scenarios. Modeling SCA led to small increases in prediction accuracy for GY and PH, with a maximum increase in the T0 scenario (Table S1).

### Prediction accuracy for novel single-cross family

**Figure 4:**
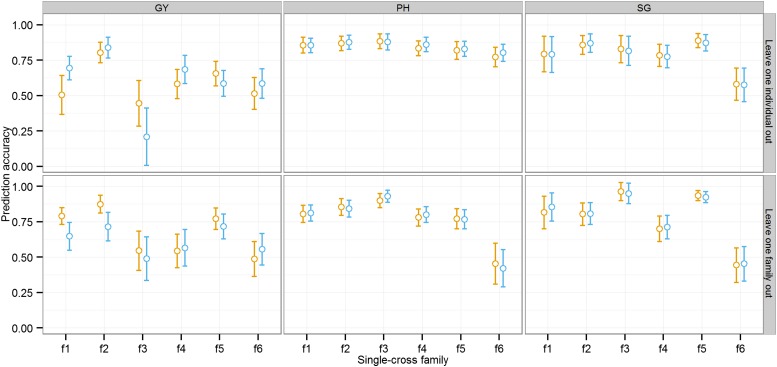
Mean prediction accuracy and SE of methods 1a (orange) and 1b (blue) in predicting performance of novel single-cross families. Two cross-validation schemes were used: leave-one-family out (bottom panel) and leave-one-individual out (top panel). Traits analyzed were grain yield (GY), plant height (PH), and staygreen (SG). SEs were estimated using the bootstrap method.

We next investigated the potential to predict the performance of single crosses in a new single-cross family, using the phenotypic and genotypic information on the single crosses from related single-cross families (leave-one-family-out cross-validation). When eight of the families were used as a training set to predict performances of single crosses from the remaining family, prediction accuracies were generally moderate for GY and high for PH and SG (Figure 4). The mean accuracies of methods 1a and 1b for prediction of novel single-cross families were 0.67 and 0.62 for GY, 0.85 and 0.76 for PH, and 0.78 and 0.78 for SG, respectively (Table S2). Variation in prediction accuracy across families was observed, especially for GY. We also evaluated the effect of adding single crosses from the family being predicted to the training set by comparing prediction accuracy of individual family with leave-one-individual-out and leave-one-family-out cross-validations. The goal of this analysis was to measure the benefit of including information from the same single-cross family to accurately separate single crosses within the same family. Although the prediction accuracies were increased slightly for some families, they were decreased for other families (Figure 4). The mean prediction accuracies of methods 1a and 1b were 0.60 and 0.61 for GY, 0.84 and 0.85 for PH, and 0.79 and 0.78 for SG, respectively. Adding individuals from the family being predicted benefited method 1b more than method 1a (Table S2).

### Genomic predictions of GY of all possible single crosses

**Figure 5:**
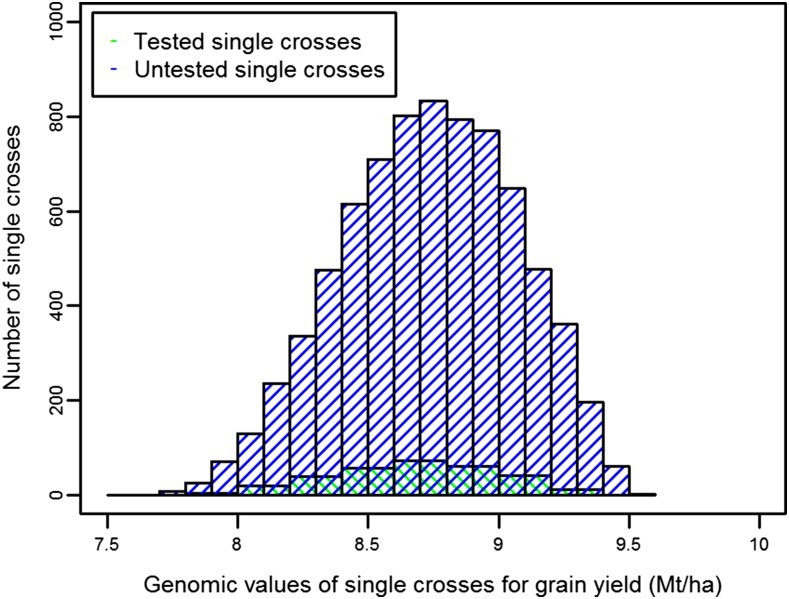
Distribution of genomic predictions for grain yield (GY) for all 7866 possible single crosses between the 46 SSS inbred progenies and 171 NSS inbred progenies.

Genomic predictions were calculated for all possible 7866 single crosses between 46 SSS and 171 NSS inbred progenies based on the prediction model, including parent GCA and cross SCA effects (*i.e.*, method 1b). The genomic predictions for GY ranged from 7.5 to 9.5 Mt/ha. The top 100 single crosses based on genomic predictions included only one single cross that was actually made and tested; the remaining 99 single crosses were never made. Moreover, >50 untested single-cross combinations surpassed the highest genomic prediction of any tested single cross (Figure 5).

## Discussion

Typical hybrid maize breeding programs involve the creation of large biparental families for topcrossing to elite testers early in the breeding pipeline. Early-stage selections are performed on the basis of topcross performance with a single elite tester, which is the sum of the candidate line GCA effect and any SCA effect between the candidate line and tester. While this is a very convenient and routine method, it has long been recognized that it would be ideal to test all combinations of possible parents immediately in the hybrid breeding pipeline (Fehr 1987). There are two main advantages of early evaluation of all potential single crosses. First, it could identify the best parental combination immediately after progeny development. Selection of inbred progenies only on the basis of topcross evaluation leaves open the possibility that some unique parental combinations never made and tested could be superior in performance and become commercial hybrids. Second, early evaluation based on single-cross performance could enable commercialization of hybrids in less time by essentially skipping the topcross stages. Despite these advantages, field testing of all potential single crosses of inbred progenies is completely impractical for a mature hybrid maize breeding program.

Advances in genotyping technology, such as GBS, have made it practical to genotype all parental candidate lines with dense, genome-wide markers (He *et al.* 2014). Genomic prediction models can predict the performance of all possible single-cross combinations, allowing *in silico* evaluation of all parental combinations, as in the ideal scenario described above. In the present study, GBS and yield trial data were used to build genomic prediction models for predicting single-cross performance. The accuracy of predicting single crosses estimated using cross-validation ranged from 0.28 to 0.77 for GY, 0.53 to 0.91 for PH, and 0.49 to 0.94 for SG, depending on the model and cross-validation scenario (Table S2). These prediction accuracies were 37–97, 56–96, and 54–100% of the accuracy of phenotypes $\left(\sqrt{h^{2}}\right)$ for GY, PH, and SG, respectively. Therefore, the prediction accuracies of single-cross performance achieved in this study indicate that this approach holds great potential for increasing the efficiency of a hybrid breeding program by enabling the effective evaluation of all single-cross combinations.

### Prediction accuracy for T2, T1F, T1M, and T0 single crosses

**Figure 6:**
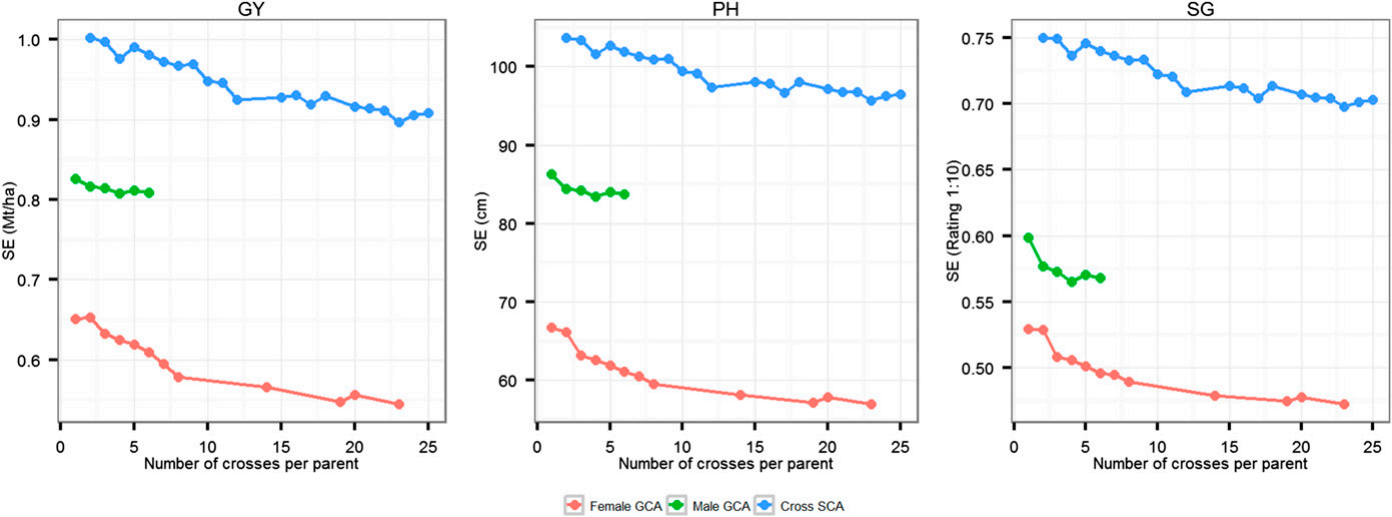
SEs of predicted GCA and SCA effects estimated using differing numbers of single crosses per parental inbred progeny.

In order to understand the effect of parent testing on the accuracy of single cross predictions, we evaluated the accuracies of prediction of single crosses having both (T2), either female (T1F) or male (T1M), or no (T0) parent tested for single-cross performance. Observed differences in prediction accuracies between these scenarios were considerable, with the highest prediction accuracy for T2 single crosses followed by T1 (T1F or T1M) and T0 single crosses. The T0 scenario was the most difficult to predict. Similar trends have been observed using simulations (Technow *et al.* 2012), as well as experimental studies based on historical data in maize (Massman *et al.* 2013; Technow *et al.* 2014) and wheat (Zhao *et al.* 2015). This finding can be explained by the representation of parents among a differing number of single-cross combinations in the training set. The information shared between the single cross being predicted and the training set increases as the number of times the parents are tested in different single cross combinations. As a result, the GCA and SCA effects are estimated with high accuracy, as indicated by decreasing SEs along with increased representation of single-cross parents in the training set (Figure 6). In the T2 scenario, both parents are tested in multiple single-cross combinations within the training set, enabling accurate estimation of parent GCA effects. With a preponderance of GCA variance over SCA variance, genotypic values of T2 single crosses can, therefore, be predicted with higher accuracy. In the T1 scenario, however, only one of the parents is tested in single-cross combination and consequently the prediction accuracy of T1 single crosses is lower than for T2 single crosses. The mean of T1 single-cross prediction accuracies observed in this study were 78, 86, and 80% of the T2 single-cross prediction for GY, PH, and SG, respectively. The prediction accuracy of the T1F single crosses is greater than that of the T1M single crosses. This finding can be explained by the smaller total number of females than males, which increases the number of times each female is tested in a single-cross combination. The mean accuracies of T0 single-cross prediction were 53, 75, and 59% of the mean accuracies of T2 single-cross prediction for GY, PH, and SG, respectively. This suggests that performance of single crosses having at least one tested parent can be effectively predicted using genomic-estimated GCA and SCA effects, but prediction accuracies suffer considerably if neither of the parents of a single cross are tested. This issue should be studied using larger population sizes, both in terms of more interconnected biparental populations and progenies per population, to determine if population size can overcome parent representation in the training set.

### Comparison of single-cross prediction methods

The published studies on prediction of single-cross performance have used covariance among tested and untested single crosses (methods 2a and 2b) to predict the performance of untested single crosses (Massman *et al.* 2013; Technow *et al.* 2014). In an alternative approach, we used genomic-estimated GCA and SCA (methods 1a and 1b) to predict the performance of untested single crosses. The comparison of prediction accuracies showed that the four methods achieved comparable accuracies for predicting T2 and T1F single crosses. For T1M and T0 single crosses, however, methods 1a and 1b provided higher prediction accuracies compared with methods 2a and 2b. Although the same information is input into the two different types of methods (*i.e.*, additive genomic relationship matrices of parents, dominance relationship matrix of the single crosses), the methods differ in their underlying assumptions and in the way in which resulting predictions are calculated. Methods 1a and 1b use the three genomic relationship matrices separately in order to model the covariances of GCA effects of females, males, and SCA effects of single crosses. Methods 2a and 2b, on the other hand, combine the covariance matrices to estimate the single-cross covariance through summation of the covariance between the female parents and the covariance between the male parents. This difference can explain the improved performance of method 1 over method 2 under the T1M and T0 cross-validation scenarios. In general, the amount of information used from the tested genotypes for BLUP of untested genotypes depends upon the covariance between the tested and untested genotypes. The genetic covariance is a function of the population genetic variance and genetic relationship. In the case of a single population, the genetic variance is constant and thus, in essence, most of the information used in calculating BLUP of an untested genotype is derived from the most closely related tested genotypes. However, in the case of two separate populations, two individuals with higher covariance from the first population would not necessarily be more closely related than two individuals with a lower covariance from a second population. This is due to the fact that the genetic covariance estimate is population specific, as it depends on the genetic variance within a population (Falconer and Mackay 1996). As a result, in single crosses where two parental populations are involved (female population and male population), having higher covariance (as estimated in this and previous studies) may not mean they are more related than single crosses having lower covariance. Methods 2a and 2b, however, invariably assume that single crosses having higher covariance are more related than single crosses with lower covariance. This could result in wider use of information from comparatively less related single crosses, which could ultimately affect prediction accuracy.

To test this hypothesis for differences in prediction accuracy of two groups of methods, we randomly sampled a balanced (equal number of females and males) subset of single crosses among 40 females and 40 males. Methods 2a and 2b were modified to correct the discrepancies in weighting of relationship between females and males by using the average of GCA variance of females and males. Specifically, for method 2a, the elements of $C_{ut}$ and off-diagonal elements of $C_{tt}$ were computed as:

$${\left(G_{f}\right)}_{ii^{\prime }}\left(\frac{\sigma_{GCA\_F}^{2}+\sigma_{GCA\_M}^{2}}{2}\right)+{\left(G_{m}\right)}_{jj^{\prime }}\left(\frac{\sigma_{GCA\_F}^{2}+\sigma_{GCA\_M}^{2}}{2}\right).$$

The diagonal elements of $C_{tt}$ were estimated as:

$${\left(G_{f}\right)}_{ii}\left(\frac{\sigma_{GCA\_F}^{2}+\sigma_{GCA\_M}^{2}}{2}\right)+{\left(G_{m}\right)}_{jj}\left(\frac{\sigma_{GCA\_F}^{2}+\sigma_{GCA\_M}^{2}}{2}\right)+\sigma_{\bar{X}}^{2}.$$

Method 2b was similarly modified. The GY prediction accuracy of the four methods for the T2, T1F, T1M, and T0 scenarios was evaluated using leave-one-individual-out cross-validations. The modified methods 2a and 2b obtained higher accuracies for T1M and T0 compared with the original methods 2a and 2b (Table 4). The accuracies were comparable to methods 1a and 1b. For T2 and T1F scenarios, however, the modified methods 2a and 2b obtained lower accuracies than the original methods. This could also be explained based on our hypothesis. The use of average GCA variance enabled the model to extract information from more closely related tested single crosses. However, the amount of information extracted is related to the genetic covariance, which, as mentioned above, is a function of the genetic relationship and genetic variance. The T2 and T1F scenarios include tested female parents in the training set. Because GCA variance of the female population is larger in our study, use of the average GCA variance lowered the amount of information from the female parents relative to what would have been achieved if the populations were treated separately.

**Table 4:** Correlation between observed and predicted grain yield (GY) in a random balanced subset of hybrids for three groups of hybrid prediction methods, as evaluated by leave-one-individual-out cross-validation

Hybrid	Prediction Methods					
	1a	2a	2a*^a^*	1b	2b	2b*^a^*
T0	0.185	0.136	0.201	0.197	0.144	0.196
T1M	0.200	0.147	0.180	0.071	0.111	0.161
T1F	0.449	0.422	0.378	0.449	0.422	0.374
T2	0.445	0.419	0.315	0.336	0.346	0.301

a Modified method.

Overall, these results indicate that single-cross covariance-based methods (2a and 2b) confound the genetic relationship and genetic variance in using information from tested single crosses. The combining ability-based methods (1a and 1b) correctly use genetic relationships and variances by separately estimating female and male GCA. The previous studies on single-cross prediction in maize (Schrag *et al.* 2009, 2010; Technow *et al.* 2014), wheat (Longin *et al.* 2013), sunflower (Reif *et al.* 2013), and triticale (Gowda *et al.* 2013) have reported different estimates of GCA variance between two parental populations of single crosses. This suggests that single-cross prediction based on genomic estimated GCA and SCA is a better approach compared with the commonly used method using genomic covariances among single crosses.

The prediction accuracies for T2, T1, and T0 single crosses reported by Technow *et al.* (2014) and T2 and T1 single crosses reported by Massman *et al.* (2013) are higher than the corresponding accuracies observed in the present study. This discrepancy is likely due to differences in population and family structure between the present study and those previously reported. Massman *et al.* (2013) and Technow *et al.* (2014) used single crosses made among a diverse set of established inbred parents that could be clustered into genetic groups whereas the present study consisted of many inbred progenies from only three families on each side of the heterotic pattern. Moreover, the families shared parents, resulting in less variation among families. As Windhausen *et al.* (2012) reported, when population structure is present and not accounted for, the prediction accuracy can derive mostly from differences in mean performances between subpopulations. Our population contained less genetic variation and fewer subpopulations compared with previously reported studies. In addition, the average number of single-cross combinations per parental line was higher in previously reported studies.

### The benefit of modeling SCA

We observed an increase in single-cross prediction accuracy for GY by modeling and estimating SCA effects and subsequently summing GCA and SCA effects. The increase in accuracy by modeling SCA was highest for T0 single crosses, followed by T1 and T2 single crosses. This result suggests that modeling SCA can be more beneficial for single crosses with untested parents compared with those with one or two tested parents. These findings could possibly be explained by a small number of single-cross combinations per parental line. If parents are tested in a small number of single-cross combinations, as in the present study, their GCA effect predictions could capture a significant portion of the SCA effect as well. The increase in prediction accuracy achieved by adding SCA would clearly depend on the magnitude of the SCA bias of the predicted GCA effect. We do not have the ability to estimate this bias, but in our study the ratio of SCA *vs.* GCA variance was small for all traits (Table 3). When a parent has no performance data in single-cross combination, its GCA is predicted based on all tested relatives, resulting in a predicted GCA effect less biased by SCA. Hence, SCA is expected to improve the predictions for single crosses with untested parents.

When predicting the GY of single crosses from a novel family (*i.e.*, leave-one-family-out cross-validations), the accuracy was similar or slightly lower when both GCA and SCA were included compared with GCA only. When information is not shared between family members, SCA is determined from single-cross combinations of relatives in other families, which are expected to be less accurate as SCA depends on specific parental combinations. This suggests that information from closely related single crosses is beneficial to SCA effect estimation, and that if this information is not available overall prediction accuracy can be reduced through error in the SCA effect estimation. Previously reported studies on hybrid genomic prediction in maize (Bernardo 1994), wheat (Zhao *et al.* 2013), triticale (Gowda *et al.* 2013), and sunflower (Reif *et al.* 2013) reported a small decrease in prediction accuracy by modeling an SCA effect. These studies used diversity panels as their experimental material. This decrease in prediction accuracy could be attributed to an inability to accurately predict SCA from distantly related single-cross combinations. The accuracy of SCA effect estimation should be tested in every unique situation (*e.g.*, within-family selection *vs.* selection across families) and population where single-cross genomic prediction is being used, in order to prevent a possible detrimental outcome by adding SCA effects.

### Prospects for early-stage single-cross prediction

Overall, this study indicates that breeders should consider redesigning hybrid breeding programs to take advantage of genomic prediction. The early stages of maize hybrid development consist of the generation of RILs or DHs from biparental families on each side of a heterotic pattern, followed by evaluation of their potential to serve as parents of hybrids. Traditionally, initial selections are conducted on the basis of topcross tests. Single crosses among selected inbred progenies are evaluated in later stages in the breeding pipeline. While this method has many advantages, one major disadvantage is that not all potential single crosses among breeding lines can be evaluated. Moreover, the addition of multiple years of topcross testing increases the time to hybrid release. The use of genomic prediction to identify superior single crosses could both shorten the length of time to hybrid release, and prevent the discarding of superior single crosses that just never happened to be phenotypically evaluated in the topcross system. We believe this can be achieved given the high prediction accuracies observed when both parents (T2) are included in the training set. Additionally, opportunity exists to optimize the genomic prediction of early-stage single crosses. Pedigree selection and frequent use of successful parents creates a family structure within typical hybrid maize breeding programs consisting of interconnected biparental families. The results from this study demonstrate that single-cross genomic prediction methods even hold potential for separating single crosses from a common family background (Figure 4). The prediction accuracy for novel single-cross families was moderate to high, and the addition of single crosses from the same family to the training set only minimally improved accuracy. Further study of the optimization of larger training sets through leveraging family structure could further improve the accuracy of genomic prediction of single crosses.
